# Synergistic hydrolysis of filter paper by recombinant cellulase cocktails leveraging a key cellobiase, Cba2, of *Cellulomonas biazotea*

**DOI:** 10.3389/fbioe.2022.990984

**Published:** 2022-09-28

**Authors:** Faiza SIDDIQUE, Edward Kat Hon LAM, Wan Keung Raymond WONG

**Affiliations:** ^1^ Division of Life Science, The Hong Kong University of Science and Technology, Clear Water Bay, Kowloon, Hong Kong, China; ^2^ Green Faith (International) Environmental Technology Ltd, Unit G, 19/F, King Palace Plaza, Kwun Tong, Kowloon, Hong Kong, China

**Keywords:** *Cellulomonas biazotea*, *Cellulomonas fimi*, cellobiases, endoglucanases, exoglucanases, filter paper assay, recombinant cellulases, synergistic effects

## Abstract

*Cellulomonas biazotea*, a Gram-positive cellulolytic bacterium isolated from soil, is capable of producing a complete cellulase complex exhibiting endoglucanase, exoglucanase, and cellobiase activities. Despite the presence of a full complement of all three types of cellulases, samples prepared from both cell lysates and culture media of *C. biazotea* showed only weak synergistic activities formed among the cellulase components, as reflected by their inefficient performance in filter paper hydrolysis. However, when the five previously characterized recombinant cellobiases of *C. biazotea* were mixed individually or in different combinations with recombinant enzyme preparations (CenA/Cex) containing an endoglucanase, CenA, and an exoglucanase, Cex, of another *Cellulomonas* species, *C. fimi*, the cellulase cocktails exhibited not only much higher but also synergistic activities in filter paper hydrolysis. Among the 5 *C. biazotea* cellobiases studied, Cba2 was shown to perform 2.8 to 3.8 times better than other homologous isozymes when acting individually with CenA/Cex. More noteworthy is that when Cba2 and Cba4 were added together to the reaction mixture, an even better synergistic effect was achieved. The filter paper activities resulting from Cba2 and Cba4 interacting with CenA/Cex are comparable to those obtained from some commercial fungal cellulase mixtures. To our knowledge, our results represent the first demonstration of synergistic effects on filter paper hydrolysis achieved using recombinant bacterial cellulases.

## 1 Introduction

The development of a cost-effective process for producing ethanol biofuel using cellulosic residues derived from agricultural, forestry, and municipal sources as substrates has been an intriguing and challenging research topic (Ragauskas et al., 2006). Strategies including co-administration of cellulose saccharification and microbial fermentation (Blondin et al., 1983; Xin et al., 2019; Dadwal et al., 2021), co-expression of heterologous cellulase genes in the same yeast host in attempting to achieve simultaneous substrate saccharification and fermentation (Wong et al., 1988), and co-cultivation of cellulolytic and fermentative strains to bring out augmented fermentation performance (Maki et al., 2009) have all been proposed as ways to accomplish the mission.

The advent of a practical approach to cellulolysis, which involves collaborative action among three different types of cellulases: endoglucanases (EC 3.2.1.4), exoglucanases (EC 3.2.1.91), and cellobiases (EC 3.2.1.21), is the biggest stumbling block to the production of cellulosic ethanol. In the hydrolytic process, cellobiose presents strong inhibition to both endoglucanase and exoglucanase activities, which are, however, less susceptible to the inhibition exerted by the final product, glucose (Gao et al., 2017). Thus, to achieve synergy in cellulolysis, the presence of a compatible cellobiase component to work with other cellulases is apparently indispensable (Singhania et al., 2017).

Cellulases of fungal sources are commonly found to exhibit potent cellulolytic activities (Nathan et al., 2014; Gao et al., 2017), reflecting among them the presence of a full complex of all three types of cellulases which act to result in synergistic hydrolysis. Unfortunately, due to the co-existence of large amounts of heterogeneously unrelated host proteins, cellulases obtained from native fungal organisms are in general low in specific activities, which are inadequate for direct application to cellulolysis. On the other hand, cross synergism has also been shown to exist among cellulases derived from different microbial species (Wood and Mccrae, 1979; Woodward, 1991; Himmel et al., 2018). Despite this alternative choice, the extent of synergism among heterologous cellulases depends upon the genetic distance among the organisms from which the enzymes are derived, the composition of the cellulase mixture, and the complexity of the substrate to be hydrolyzed (Wood and Mccrae, 1982; Yang et al., 2018).

Another challenge to the implementation of a cellulolytic process for practical application is the capability of producing various cellulase components on a large scale. The emergence of recombinant DNA technology in the 1970s has facilitated the research and development of a scalable protocol for cellulose processing. Since the cloning and characterization of a bacterial cellulase gene were first achieved in the early 1980s (Whittle et al., 1982), a wide collection of structural genes encoding all three types of cellulases have been cloned, expressed, and characterized from a diversity of microbial species (Shoemaker et al., 1983; Gilkes et al., 1984a; Gilkes et al., 1984b; Skipper et al., 1985; Johnson et al., 1986; O’Neill et al., 1986; Wong et al., 1986; Arsdell et al., 1987; Wong et al., 1988; Barnett et al., 1991; Lam et al., 1997; Wong et al., 1998; Thapa et al., 2020). Through the studies, vast amounts of data have been gathered on the sequences of various cellulase genes, as well as the functional and structural properties of the encoded cellulase products. On the other hand, genetic engineering has enabled the development of innovative strategies and expression systems for the efficient production of heterologous cellulases (Skipper et al., 1985; Wong et al., 1988; Lam et al., 1997; Lam et al., 1998; Fu et al., 2006; Wang et al., 2010; Wang et al., 2011; Wong et al., 2012; Kwong et al., 2016a; Singh et al., 2017; Hu et al., 2018; Wong et al., 2019). The availability of recombinant approaches for cellulase expression does not only facilitate the engineering of feasible tactics and platforms for the large-scale production of cellulases (Juturu and Wu, 2014; Bhati et al., 2021), but also the formulation of enzyme mixtures for the performance of synergistic studies (Wang and Lu, 2016).

Our group has been involved in the research on applications of recombinant cellulases since the mid-1980s. We pioneered the development of various microbial systems and strategies with the aims to: 1) facilitate the cloning and expression of target cellulase genes; 2) provide a competent surrogate host(s) for the expression and/or co-expression of cellulases; 3) achieve enhanced levels of production of cellulases for co-operative studies (Skipper et al., 1985; Wong et al., 1988; Lam et al., 1997; Lam et al., 1998; Fu et al., 2006; Wang et al., 2010; Wang et al., 2011; Chan et al., 2012; Wong et al., 2012; Kwong et al., 2016a; Chan et al., 2018; Wong et al., 2019; Siddique et al., 2021). Previously, making use of an enzyme mixture comprising an endoglucanase, CenA, and an exoglucanase, Cex, of *C. fimi* prepared from a common yeast host, we were able to demonstrate the existence of cooperativity formed between CenA/Cex and a commercial cellobiase preparation in cellulose degradation (Wong et al., 1988). Nonetheless, the cooperative activities were shown to be weak and inadequate for commercial applications.

Understanding that cellulases derived from closely related species have a greater likelihood than those from distant relatives to form synergism in cellulolysis, we started to clone cellobiase genes from a close relative of *C. fimi*, *C. biazotea*, which is not only fully cellulolytic but also capable of producing secretory cellobiase activities to the culture medium (Lau and Wong, 2001). After many years of laborious undertaking, we succeeded in cloning five different cellobiase genes from *C. biazotea* (Wong et al., 1998; Chan et al., 2012; Chan et al., 2018; Siddique et al., 2021), which might represent the full complement of genetic determinants for cellobiases in the organism. These five cellobiase genes and their encoded products have been well characterized (Wong et al., 1998; Chan et al., 2012; Chan et al., 2018; Siddique et al., 2021). Although all five cellobiases were found to be secretory when expressed in either *C. biazotea* (Cba2; Lau and Wong, 2001) or *E. coli* (the remaining four members; Wong et al., 1998; Chan et al., 2012; Chan et al., 2018), interestingly, only one of them, Cba2, was confirmed recently to possess a typical Sec pathway signal peptide, supporting the interpretation that Cba2 is a truly secretory protein, which has so far been the first bacterial cellobiase demonstrated to bear a genuine secretion signal peptide (Siddique et al., 2021). In this communication, we report the engineering of various bacterial platforms to productively express the 5 *C. biazotea* cellobiases, CenA and Cex, leveraging our better understanding of both the expression tactics and cellulase characteristics. We then present the results obtained from a systematic study investigating whether or not synergies occur among the recombinant cellulases in filter paper hydrolysis. Our data provide evidence to support the conclusion that the cellulase cocktails work cooperatively to achieve synergistic effects on the degradation of filter paper. Despite the positive outcome, it is noteworthy that only some of the cellobiase components are capable of collaborating with CenA and Cex to result in practically valuable synergies. Our results demonstrate, for the first time, the applicability of a bacterial cellobiase, Cba2, to the achievement of synergies with other types of cellulases, and more interestingly, with another cellobiase, Cba4, in hydrolyzing filter paper. In view of the high levels of synergistic activity attained, the heterologous production approach reported in this work may shed light on the development of a scalable process utilizing recombinant cellulases for the hydrolysis of complex cellulose.

## 2 Materials and methods

### 2.1 Bacterial strains, chemicals, and media

*E. coli* JM101 (Sivakesava et al., 1999) *and B. subtilis* 1A751 (Kwong et al., 2013) were used for DNA manipulations. 2x YT and 2x LB media were used for the cultivation of *E. coli* (30°C) and *B. subtilis* (37°C) cultures, respectively (Wong et al., 1998; Ng et al., 2016). *C. biazotea* was cultivated in 1.2 L 2x YT + 1% low viscosity CMC culture medium for 48 h (Lau and Wong, 2001). After centrifugation, the supernatant was retained and the pellet was lysed using a pressure cell homogenizer (Ng et al., 2016). The supernatant was concentrated 5 times. Both the cell lysate and concentrated supernatant were clarified by centrifugation.

The Phusion PCR Kit, restriction and modifying enzymes were purchased from NEB (Ipswich, MA, United States). All oligos were purchased from Invitrogen (Carlsbad, CA, United States). Chemicals used in this study were purchased from Sigma-Aldrich Corporation (St. Louis, MO, United States) unless otherwise specified. A commercial β-glucosidase of almond origin (Sigma Cat No. G-0395) was employed in this study.

### 2.2 Expression of recombinant cellulases

Plasmid constructs: pMBH6cenA-deltaA encoding CenA (Sze, 2011; unpublished results); pM1VegGcexL encoding Cex (Fu et al., 2006); pMBH6cbap encoding Cba (Chan, 2012); pM2VegCEGFDnaBcba2 encoding Cba2 (this study); pMBcba3 encoding Cba3 (Chan et al., 2018); pMBcba4b encoding Cba4 (Chan et al., 2018); pMcba5b-B2 (ATG) encoding Cba5 (Chan et al., 2018) were engineered previously or in this study. Growth conditions for the cultivation of *E. coli* transformants harboring the above constructs (except Cba2) were described previously (Fu et al., 2006; Chan et al., 2012; Chan et al., 2018). For the cultivation of the *B. subtilis* transformant expressing Cba2, the medium was supplemented with 20 μg ml^−1^ of kanamycin. After the growth at 250 rpm and 37°C had reached an A_600_ value of 1.0, a final concentration of 0.2 mM IPTG was added to the growing cells. Culture samples were then collected and the cells were lysed using a French press (Lam et al., 1997). The clarified lysates were then lyophilized and used for setting up assays.

### 2.3 Construction of pM2VegCEGFDnaBcba2

**FIGURE 1:**
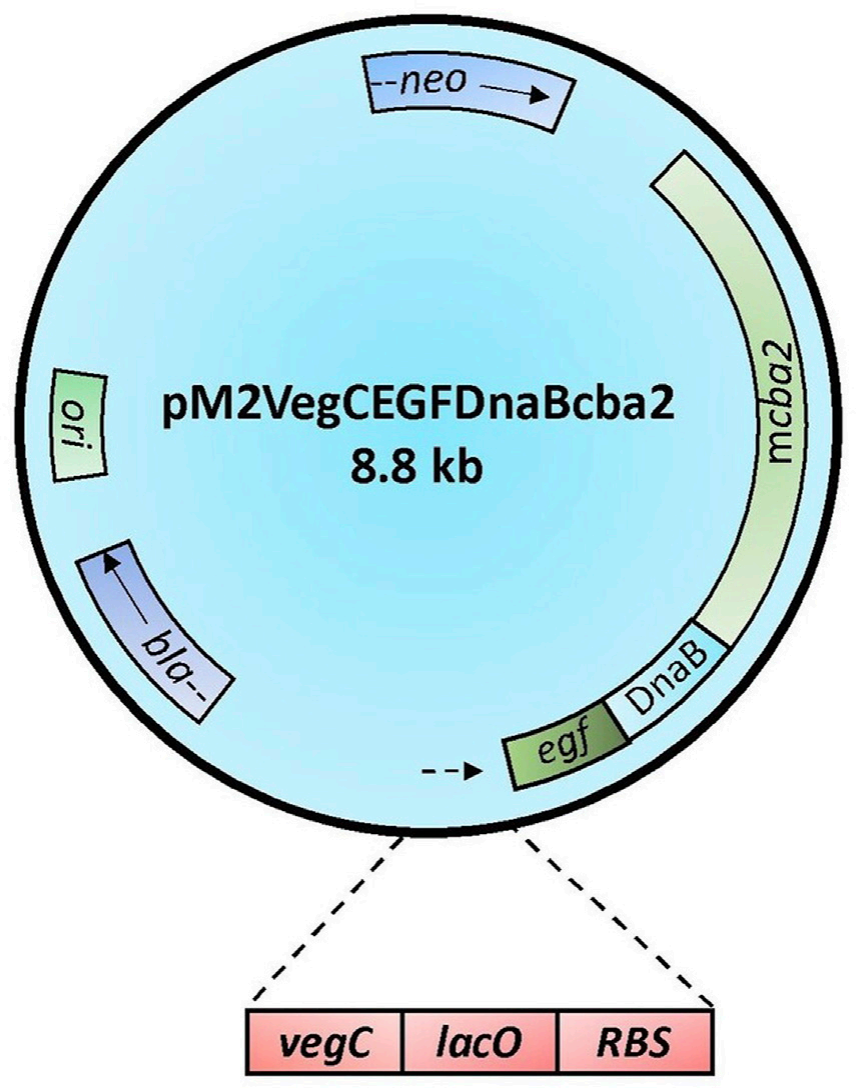
Schematic representation of construct pM2VegCEGFDnaBcba2. Symbols for genetic elements are as follows: *egf* = human epidermal growth factor gene; DnaB = coding sequence of *Ssp* DnaB intein; m*cba2* = cba2 gene expressing mature Cba2; *neo* = structural gene conferring resistance to neomycin; *ori* = origin of replication in *B. subtilis*; *bla* = β-lactamase gene. The lower panel shows the regulatory elements of the Veg expression cassette comprising the VegC promoter (*vegC*)*,* the *lac* operator (*lac*O), and the consensus ribosome binding site in *B. subtilis* (RBS). The arrows indicate the directions of transcription.

The construction of plasmid pM2VegCEGFDnaBcba2 (Figure 1), which employed the DnaB intein (Hu et al., 2018) to mediate Cba2 expression in *B. subtilis*, relied on the application of DNA constructs previously engineered in our laboratory for expressing various heterologous proteins to: 1) achieve the formation of a precursor DNA fusion comprising coding sequences for the following genetic elements: *Eco*RI-*veg*C-*lac*O-RBS-EGF-DnaB-*cba2*; 2) serve as a vector to mediate Cba2 expression wherefrom the precursor fusion protein was subjected to auto-cleavages to facilitate the separation between Cba2 and the DnaB fusion partner in *B. subtilis*.

There were altogether three major steps involved in the development of the long DNA fusion product mentioned in point 1) above. Firstly, employing construct pM2-CellBD-DnaB-bFGF (Hu et al., 2018) as the template and oligos P1 and P2 (Table 1) as the primers, a PCR was performed to obtain a 113-bp product, *Eco*RI-*veg*C-*lac*O-RBS, comprising an *Eco*RI restriction site, along with the following regulatory components: *vegC* promoter and *lac* operator of *E. coli*, and RBS of *B. subtilis* (Lam et al., 1998). Secondly, aiming to achieve a fusion product: RBS-EGF-DnaB-*cba2*, another PCR in which an intermediate construct, pWK3R-EGF-DnaB-bFGF (Hu et al., 2018), and oligos P3 and P4 (Table 1) were employed as the template and primers, respectively, was undertaken. Lastly, a partial *cba2* gene sequence spanning from the 7^th^ (GGC) codon to the 127th codon (part of the unique *Stu*I site on the *cba2* sequence) encoding the N-terminal mature Cba2 product was generated through the 3^rd^ PCR employing the *cba2* gene carried on construct pMcba2 (Siddique et al., 2021) as the template and oligos P5 and P6 (Table 1) as the primers.

**TABLE 1:** Primers employed in the construction of plasmid pM2VegCEGFDnaBcba2.

Primer^a^	Sequence^b^
P1: *Eco*RI-VegC F	5′-GGG​gaa​ttc​TAA​TTT​AAA​TTT​TAT​TTG​ACA​AAA​ATG​GGC​TCG​TGT​TGT​CCA​ATA​AAT​GT-3′
P2: VegC-lacO-RBS R	5′-CAT​TTT​TAT​CAC​CTC​CTT​TGT​GAA​ATT​GTT​ATC​CGC​TCA​CAA​TTC​CAC​CTC​ACT​ACA​TTT-3′
P3: RBS-EGF F	5′-GAG​GTG​ATA​AAA​ATG​AAT​AGT​GAC​TCT​GAA​TGT​CCC​CTG​TCC​CAC​GAT​GGG​TAC​TGC​CTC-3′
P4: DnaB-cba2 R	5′-CGC​CTC​GCC​GTC​CGG​GTC​GTC​GCC​TGC​GTT​GTG​TAC​AAT​GAT​GTC​ATT​CGC​GAC​AAA-3′
P5: cba2 F	5′-GCG​AGG​CGG​CCG​TCA​CCG​AGC​GGC​TGC​AGC​AGG​GGC​GGT​CGC​TGC​CG-3′
P6: cba2-*Stu*I R	5′-CGG​Aag​gcc​tCG​TCG​CCT​GCC​ACC​GTC​CCG​TAC​TGC​TCC​GCG-3′

a F and R stand for forward and reverse, respectively.

b Restriction sites used in the cloning experiments are shown in lowercase.

The three overlapping PCR products were then subjected to an OE-PCR employing P1 and P6 as primers to obtain a fragment of 1117-bp extending from the 5’ *Eco*RI-*veg*C-*lac*O-RBS-EGF-DnaB sequence to the 127th codon (location of the *Stu*I site) of *cba2* (Siddique et al., 2021).

Both the 1117-bp product and vector pM2VegGspacba2, which was previously engineered to mediate secretory Cba2 expression in *B. subtilis* (Ng, 2013), were restricted with *Eco*RI and *Stu*I, followed by ligating the PCR fragment with the larger restriction fragment of the vector to form the final expression construct, pM2VegCEGFDnaBcba2, in which the *spa* secretion leader (Lam et al., 1998) was deleted, for intracellular Cba2 expression in *B. subtilis*.

### 2.4 Enzymatic assays

#### 2.4.1 *p*NPGase assay

The *p*NPGase assay for the quantification of activities of the 5 *C. biazotea* cellobiases and the commercial β-glucosidase product hydrolyzing *p*-nitrophenyl β-d-glucopyranoside (*p*NPG) was performed as described previously (Wong et al., 1998; Lau and Wong, 2001; Chan et al., 2012; Chan et al., 2018). One unit of *p*NPGase activity is defined as the amount of enzyme capable of releasing 1 μmol of *p*-nitrophenol (*p*NP) from *p*NPG per min.

#### 2.4.2 *p*NPCase assay

The *p*NPCase assay for the quantification of exoglucanase (Exg) activities was performed as previously described (Lam et al., 1997; Fu et al., 2006). One unit of *p*NPCase activity is defined as the amount of enzyme capable of releasing 1 μmol of *p*-nitrophenol (*p*NP) from *p*NPC per min.

#### 2.4.3 CMCase assay

The quantitative determination of endoglucanase (Eng) activities in hydrolyzing carboxymethylcellulose (CMC) was carried out using the colorimetric DNS assay as reported previously (Miller et al., 1960; Gilkes et al., 1984a; Gilkes et al., 1984b). One unit of CMCase activity is defined as the amount of enzyme capable of releasing 1 μmol of glucose equivalents from CMC per min (Wong et al., 1986; Lam et al., 1998).

#### 2.4.4 Filter paper assay

Filter paper activities were determined by the IUPAC method essentially carried out according to Ghose’s protocol (Ghose, 1987; Eveleigh et al., 2009) with the following minor modification, in which different cellulase powder stocks were each resuspended in 3 ml of sodium phosphate buffer (0.1 M, pH 5.8) for activity measurements. Several dilutions of each enzyme sample were then prepared to identify the concentration capable of releasing 2 mg of glucose from 50 mg of filter paper strip (Whatman No. 1). Filter paper activity (FPU ml^−1^) was calculated as described previously (Singhania et al., 2016). One FPU is defined as the amount of enzyme capable of producing 2 mg of reducing sugar from the filter paper per min (Eveleigh et al., 2009).

## 3 Results

### 3.1 Cooperative effects exerted between CenA/Cex and various cellulase preparations on filter paper hydrolysis

**FIGURE 2:**
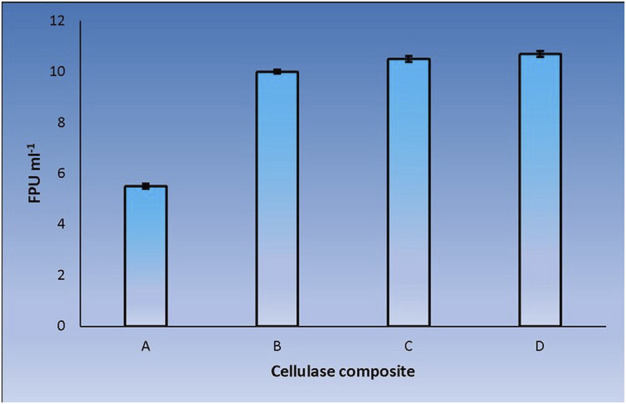
Filter paper activities (FPU) obtained from the interaction between CenA/Cex and a commercial β-glucosidase. Sample **(A)** denotes the CenA/Cex cocktail comprising 100 U CenA and 2.5 U Cex, whereas reactions **(B)**, **(C)**, and **(D)** comprised in addition to CenA/Cex, 0.001 U, 0.005 U, and 0.01 U of commercial β-glucosidase, respectively. The data shown are mean ± SEM values deduced from three independent assays.

CenA/Cex prepared from a recombinant *Saccharomyces cerevisiae* host was shown previously to provide a weak cooperative effect with a commercial β-glucosidase product on filter paper hydrolysis (Wong et al., 1988). Despite applying CenA/Cex derived from a distinctly different source, *E. coli*, the change did not seem to have a striking impact on its effectiveness in pairing up with the commercial β-glucosidase in filter paper hydrolysis (Figure 2). The findings supported the conclusion that the two sets of enzymes were derived from two distantly related organisms that failed to achieve significant synergy in cellulolysis.

Investigating along the lines of cladistics, we were attracted by the ability of *C. biazotea*, a soil bacterium, which is not only genetically close to *C. fimi*, from which CenA and Cex were identified (Whittle et al., 1982; Gilkes et al., 1984a; Gilkes et al., 1984b), but also capable of producing a full complex of cellulases, as reflected by the presence of all three types of cellulase activities in the culture samples (Table 2). Moreover, the samples displayed a notable feature which was the ability to saccharify filter paper, despite the detection of only weak activities in both the *C. biazotea* lysate and supernatant samples (Table 2). Nevertheless, the findings provided us with a useful insight into the possibility that the cellobiases from *C. biazotea* might be good candidates to interact with CenA/Cex to achieve a productive synergistic effect on cellulose degradation.

**TABLE 2:** Detection of various cellulase activities in the cell lysate and culture supernatant samples of *C. biazotea*.

	Sample Enzyme activity^a^ and (specific activity^b^ )			
	CMCase	*p*NPCase	*p*NPGase	Filter paper activity
Cell lysate	0.7725 (0.0684)	0.0715 (0.0063)	0.2589 (0.0229)	1.3 ± 0.013
Culture supernatant	0.1064 (2.448)	0.0041 (0.095)	0.0298 (0.6852)	2 ± 0.076

a The unbracketed values represent the respective enzyme concentrations (U ml^−1^) of CMCase, *p*NPCase, *p*NPGase, and filter paper activity detected in the cell lysate and culture supernatant samples of *C. biazotea*. The definitions of the various enzyme activities are provided in Section 2.4.

b The bracketed values represent the respective specific activities (U mg^−1,^ of proteins) of CMCase, *p*NPCase, and *p*NPGase.

### 3.2 Application of *E. coli* and *B. subtilis* to the production of various recombinant cellulases

To enable the employment of individual components of the *C. biazotea* cellobiase complex for synergistic studies, an initial mission undertaken was to clone and characterize their genetic determinants. In the past 2 decades, 5 genes encoding five individual cellobiases: Cba (Wong et al., 1998; Chan, 2012; Chan et al., 2018), Cba2 (Siddique et al., 2021; this study), Cba3 (Chan et al., 2012, Chan et al., 2018), Cba4 (Chan et al., 2018), and Cba5 (Chan et al., 2018) have been cloned and characterized by our group. Concurrently, we have also been actively involved in the engineering of various *E. coli* and *B. subtilis* expression systems applicable for the production of various recombinant proteins (Lam et al., 1998; Sivakesava et al., 1999; Fu et al., 2006; Wang et al., 2011; Wong et al., 2012; Kwong et al., 2013; Kwong and Wong, 2013; Kwong et al., 2016a; Kwong et al., 2016b; Hu et al., 2018; Wong et al., 2019). Exploiting appropriately selected *E. coli* systems, Cba, Cba3, Cba4, and Cba5 as well as CenA and Cex were produced as recombinant proteins in adequate quantities for use in synergistic studies (Table 3).

**TABLE 3:** Enzymatic activities of various recombinant cellulase preparations.

Cellulase	Total enzyme activity (U)	Enzyme activity (U ml^−1^)	Specific activity (U mg^−1^)
CenA	23,995,000	47.99	115.19
Cex	96,000	0.48	0.04
Cba	9,056,000	11.32	4.78
Cba2	0.07512	0.0000378	0.000021
Cba3	80,000	0.80	0.17
Cba4	96,000	0.96	0.08
Cba5	8,400	0.42	0.07

Cba2, however, being a large protein composed of 882 aa residues (Lau and Wong, 2001; Siddique et al., 2021), was found to be expressed in *E. coli* with difficulties. Probably resulting from both its large size and rather insoluble properties (Siddique et al., 2021), recombinant Cba2 tends to form inclusion bodies in *E. coli* (Siddique et al., 2021). Fortunately, the availability of both *B. subtilis* secretion and cytoplasmic systems (Lam et al., 1998; Hu et al., 2018) from our laboratory provided alternative means for use to express Cba2. Realizing that secretory expression of large proteins might cause adverse effects on protein passage through the secretion pathway (Lam et al., 1997; Fu et al., 2005), an intracellular approach utilizing an intein to mediate protein expression in *B. subtilis* (Hu et al., 2018) was adopted to facilitate the production of soluble recombinant Cba2. As described in Materials and Methods (Section 2.3), a plasmid construct, pM2VegCEGFDnaBcba2 (Figure 1), containing the coding sequence for the DnaB intein (DnaB) (Esipov et al., 2008), was engineered to express presumably initially an EGF-DnaB-Cba2 precursor, which was then subjected to autocatalytic cleavage to result in bioactive Cba2 in *B. subtilis*.

### 3.3 Cooperative cellulolytic activities exhibited by cross-species cellulases

**FIGURE 3:**
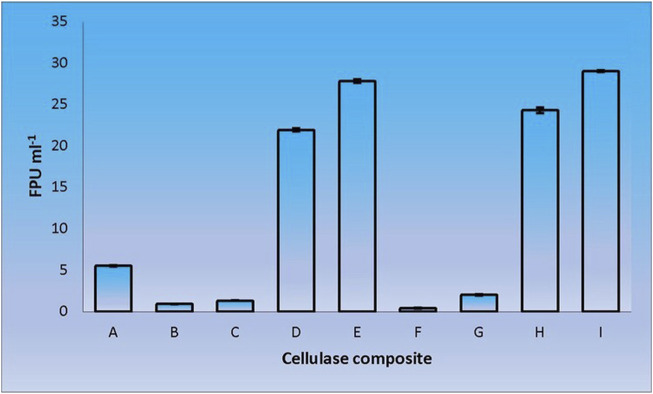
Filter paper activities (FPU) obtained from the interaction of CenA/Cex with *C. biazotea* cell lysate (CL) and culture supernatant (SN) samples. The results shown are: **(A)** CenA/Cex containing 100 U of CenA and 2.5 U of Cex; **(B)** 10 mg CL; **(C)** 25 mg CL; **(D)** CenA/Cex and 10 mg CL; **(E)** CenA/Cex and 25 mg CL; **(F)** 10 mg SN; **(G)** 25 mg SN; **(H)** CenA/Cex and 10 mg SN; **(I)** CenA/Cex and 25 mg SN. The data shown are mean ± SEM values deduced from three independent assays.

**FIGURE 4:**
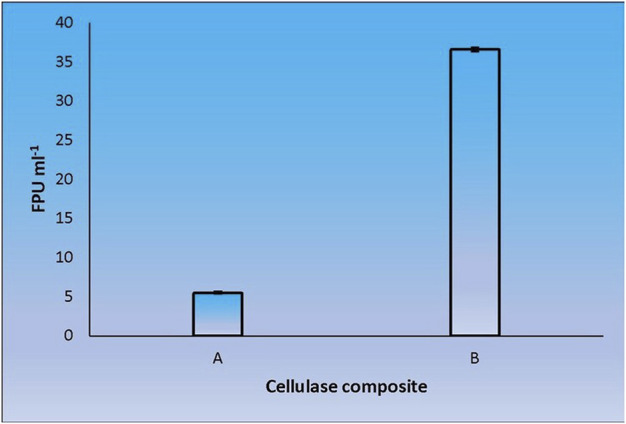
Filter paper activities (FPU) obtained from the interaction between CenA/Cex and the five cellobiase isozymes of *C. biazotea*. The results shown are: **(A)** CenA/Cex containing 100 U of CenA and 2.5 U of Cex; **(B)** CenA/Cex supplemented with 0.001 U of each of the five cellobiase isozymes: Cba, Cba2, Cba3, Cba4, and Cba5 of *C. biazotea*. The data shown are mean ± SEM values deduced from three independent assays.

When CenA/Cex was added to the cell lysate (CL) and culture supernatant (SN) samples of *C. biazotea*, it was notable that the overall activities of filter paper hydrolysis markedly increased. Although only arbitrary quantities of CenA/Cex and bacterial supernatant or lysate sample were used, over 5 to 10 folds of improvement of filter paper activities (FPU) relative to that displayed by CenA/Cex, CL or SN sample alone were detected (Figure 3). Moreover, surprisingly, when a mixture of all five of the *C. biazotea* cellobiase components, each of which conferred a small quantity of only 0.001 unit of *p*NPGase activity, was added to CenA/Cex, a remarkable enhancement of over six folds of FPU was observed (Figure 4). These findings strongly supported the idea that some, if not all, of the *C. biazotea* cellobiase components were capable of acting synergistically with CenA/Cex on cellulolysis.

### 3.4 Identification of cellobiase components capable of creating synergy with CenA/Cex

**FIGURE 5:**
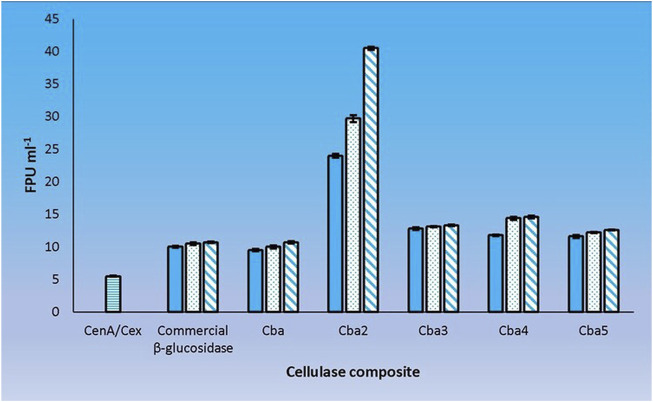
Cooperation between CenA/Cex and individual cellobiases in filter paper hydrolysis. Column CenA/Cex denotes the CenA/Cex cocktail comprising 100 U CenA and 2.5 U Cex. The rest of the columns denoted by specific cellobiase names contained, in addition to CenA/Cex, different doses: 0.001 U (plain column), 0.005 U (dotted column), and 0.01 U (column with oblique lines) of the corresponding cellobiases. The data shown are mean ± SEM values deduced from three independent assays.

A definitive way to evaluate the cooperativity between CenA/Cex and the 5 *C. biazotea* cellobiase components in filter paper hydrolysis was to assess the performance of the cellobiases individually with CenA/Cex. Our findings revealed that all five cellobiases exhibited a favorable effect with CenA/Cex on filter paper hydrolysis (Figure 5). However, the extent of cooperativity resulting from the various assortments was strikingly different, ranging from a decent improvement of around a 100% increase in FPU as shown in the mixtures individually supplemented with Cba and the commercial β-glucosidase product (Figures 2, 5), to significantly better performance (an additional 20–40% improvement) found in the combinations respectively containing Cba3, Cba4, and Cba5 (Figure 5), to finally a remarkable 7-fold enhancement in the total activity with the presence of Cba2 (Figure 5).

The results point to the fact that Cba2 is a much more efficient enzyme than the other four isozymes in hydrolyzing cellobiose, thus facilitating an effective removal of this disaccharide from inhibiting CenA and Cex activities. Presumably, the presence of Cba2 resulted in a rapid formation of glucose and a prolonged hydrolytic action of CenA/Cex (Figure 5). Moreover, another unusual property of Cba2 is its relatively long-lasting activity (Figure 5), which could be attributable to its relatively longer lifespan and/or higher tolerance to glucose inhibition.

### 3.5 Demonstration of synergistic interactions between cellobiase components in filter paper hydrolysis

Synergistic effects on cellulose hydrolysis have been well demonstrated to occur among Eng and Exg components derived from both closely and distantly related cellulolytic organisms (Wood and Mccrae, 1979). However, an investigation of synergism at the level of cellobiose degradation has been limited by the scarce availability of genuine cellobiases. Advantageously, the availability of five cellobiase components of *C. biazotea* facilitates us to carry out studies of not only their applicability to synergistic hydrolysis with other cellulases but also the possible existence of synergism among the isozymes in hydrolyzing cellobiose, an annoyingly strong inhibitor of both Eng and Exg activities (Holtzapple et al., 1990).

**FIGURE 6:**
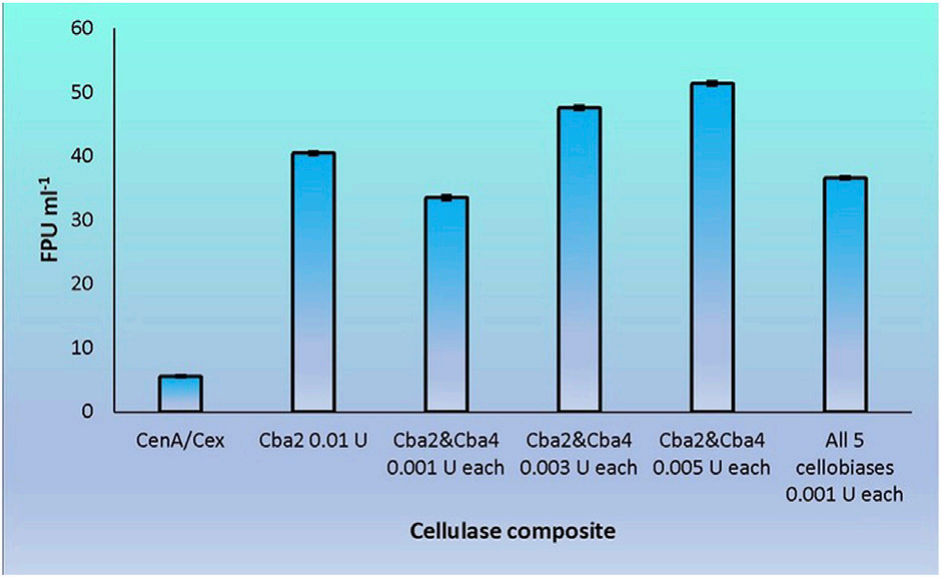
Synergistic effects on filter paper hydrolysis formed between CenA/Cex and various cellobiases. Column CenA/Cex denotes the CenA/Cex cocktail comprising 100 U CenA and 2.5 U Cex. All other columns contained, in addition to CenA/Cex, different samples and doses of cellobiases as specified. In the last reaction, all five cellobiases denote the presence of Cba, Cba2, Cba3, Cba4, and Cba5 isozymes. The data shown are mean ± SEM values deduced from three independent assays.

Cba2 was found to outperform its four competitors to yield a remarkably high score, 40.5 FPU ml^−1^, when pairing up with CenA/Cex in filter paper hydrolysis (Figure 5). On the other hand, a combination comprising all the five components, with each isozyme contributed only one-tenth of its strength (relative to that employed in individual analysis) to the cellulase mixture (Figure 5), resulted also in a notable score of 36.6 FPU ml^−1^ (Figure 6). From these observations, a couple of conclusions were drawn. First, Cba2 played an indispensable role in achieving the synergistic effects (Figure 5). Second, despite only one-tenth of the Cba2 activity (relative to that of the same enzyme acting alone) being used in the cellobiase mixture, the unexpectedly high score, 36.6 FPU ml^−1^, obtained from the teamwork (Figure 6) supported the interpretation that there should be cellobiase component(s) other than Cba2 involved in enhancing the synergistic effect.

Out of the many combinations formed between Cba2 and the other four isozymes, Cba4 was identified to be able to work with Cba2 to significantly augment the synergistic effect. When only 0.001 U of each of Cba2 and Cba4 was used in the assay, surprisingly, a considerably rewardable activity of 33.5 FPU ml^−1^ (Figure 6), which was 11% better than the performance resulting from the use of 0.005 U of Cba2 alone in the hydrolysis (Figure 5), was obtained. Interestingly, when two other combinations formed between Cba2 and Cba4, in which 0.003 U or 0.005 U of each of the two cellobiases was used, a more significantly enhanced synergistic effect was observed. The impressive outcomes of 47.6 FPU ml^−1^ and 51.4 FPU ml^−1^ were obtained (Figure 6), which were 18% and 42%, respectively, better than the activity achieved using 0.01 U of Cba2 alone in the analysis (40.5 FPU ml^−1^; Figure 5). These results provide evidence to support the conclusion that Cba2 and Cba4 work cooperatively to enhance filter paper hydrolysis. However, it is yet to be clarified how Cba2 and Cba4 exactly complement each other, in particular when higher doses of both enzymes are used, to yield better synergistic effects (Figures 5, 6). Despite working under unoptimized conditions, the FPU resulted from the cooperation between Cba2 and Cba4 (Figure 6) were shown to be comparable to those achieved by some commercial fungal cellulase preparations, ranging from 7.7 to 69.9 U ml^−1^ reported in the literature (Yu et al., 2016).

## 4 Discussion

Reconstructing an efficient cellulase complex for commercial applications has been a key research interest in our laboratory for many decades. The observation that a combination between a *C. fimi* Eng/Exg cocktail and a commercial β-glucosidase product resulting in merely narrow improvements in cellulolysis (Wong et al., 1988) strongly argued for the identification and application of a competent cellobiase component(s) if a truly constructive cellulase complex was desired. We reasoned that *C. biazotea*, being a close relative of *C. fimi* and an efficient producer of extracellular cellobiase activities (Wong et al., 1998; Lau and Wong, 2001; Siddique et al., 2021), might be a good candidate to start with. The Eng and Exg cocktail readily available for the implementation of synergistic studies with candidate cellobiases comprised CenA and Cex, which have been the first and best-characterized representatives of Eng and Exg of *C. fimi*, respectively (O’Neill et al., 1986; Wong et al., 1986). Although CenA, Cex and their isozymes were shown to be efficiently secreted to the culture medium, the co-expressed cellobiase activities were found to be cell-bound (Schimz et al., 1983; Wakarchuk et al., 1984). Therefore, it was desired that a secretory cellobiase(s) could be identified from *C. biazotea* to work cooperatively with CenA and Cex.

We have isolated five cellobiase determinants from *C. biazotea* since their first representative, *cba*, was identified and characterized in 1998 (Wong et al., 1998). Subsequently, four homologous genes including *cba3*, *cba4*, *cba5*, and *cba2* were chronologically cloned and sequenced (Chan et al., 2012; Chan et al., 2018; Siddique et al., 2021). Expression of recombinant Cba, Cba3, Cba4 and Cba5 as soluble proteins was readily achieved employing recombinant *E. coli* host systems previously engineered in our laboratory (Wong et al., 1998; Chan et al., 2012; Chan et al., 2018). However, regarding Cba2, which has recently been demonstrated to possess a genuine signal peptide to facilitate its secretory production in *C. biazotea* (Lau and Wong, 2001; Siddique et al., 2021), probably because of its large size (its mature product comprising 882 aa) and high (over 72%) GC content, its recombinant equivalent was shown to form insoluble inclusion bodies in *E. coli* (Siddique et al., 2021). Although the aggregates could be partially solubilized by adding chemical additives such as sucrose and sorbitol to the growth medium (Siddique et al., 2021), the incompletely soluble Cba2 was considered to be unsuitable for use in synergistic studies. It was then decided that *B. subtilis*, which has been engineered to offer both intracellular and extracellular expression of a wide range of bioactive recombinant proteins, might be exploited for the production of soluble Cba2. Previously, the DnaB intein (DnaB) was employed by our group to mediate the expression of authentic bFGF (Hu et al., 2018). Thus, leveraging the same strategy by fusing the *cba2* sequence encoding mature Cba2 to DnaB (Figure 1), we succeeded in expressing Cba2 intracellularly as a soluble and bioactive product in *B. subtilis*. Presumably, being also a Gram-positive bacterium, *B. subtilis* provided a more oxidizing environment than that of *E. coli* for the production of soluble Cba2 subsequent to its autocatalytic cleavage from its DnaB fusion partner.

All five recombinant cellobiases of *C. biazotea* were readily available from their hosts as intracellular lysate powders. Cytoplasmic expression offers a number of important advantages over secretory or extracellular production including the avoidance of protein transportation via the cell membrane, thus preventing unnecessary product loss through secretion/excretion. Moreover, secretory production of a heterologous protein may create detrimental effects on the cell host (Fu et al., 2005) and require extra effort and time for harvesting the product. Given the identification of a desired cellobiase candidate(s) and the availability of all the cellulase components, the pursuits of synergistic studies, scale-up production of the desired enzyme participants, and implementation of a large-scale saccharification process are expected to be readily accomplishable employing an *in vitro* approach. In addition, unlike an *in vivo* approach where endogenous cellulases of the host are present, the free of background contaminants from an *in vitro* approach facilitates synergistic studies to be undertaken conveniently and the results interpreted without complications from contaminating activities.

We adopted an *in vitro* systematic approach to examine whether there was a synergistic effect exhibited among the enzymes at two different levels: 1) between cellobaises and CenA/Cex; 2) among the five cellobiase components. The observations from level one supported the following interpretation. Firstly, as shown by the more significantly enhanced filter paper activities obtained from the mixtures of CenA/Cex with either a lysate or culture medium sample of *C. biazotea* (Figure 3) than those with the commercial β-glucosidase product (Figure 2), it was foreseeable that a mixture comprising all the five recombinant cellobiases might result in an even better synergistic effect in the comparison (Figure 4). Although the outcome emerged as expected, the five cellobiase components were found to exhibit highly different levels of efficiency in interacting with CenA/Cex. Interestingly and surprisingly, the performance of Cba2 was not only shown to be better but also much more superior, 2 to 3 times higher, than that of its counterparts (Figure 5), even though the studies were conducted under unoptimized conditions!

Cba2 was first identified as a large extracellular product of >100 kD secreted from *C. biazotea* (Lau and Wong, 2001). The enzyme was also shown to represent over 40% of total *p*NPGase activity detected in the culture medium (Lau and Wong, 2001). The ability of Cba2 to be efficiently secreted was shown and recently confirmed to be facilitated by the application of a genuine signal peptide (Lau and Wong, 2001; Siddique et al., 2021). Although the other four homologous isozymes of Cba2, which are all significantly smaller than Cba2, are all secretable, they do not possess a genuine signal peptide for secretion (Wong et al., 1998; Chan et al., 2012; Chan et al., 2018). Being a large protein conferring a major share of the cellobiase activity secreted to the surroundings of *C. biazotea*, it is understandable why Cba2 requires a signal peptide to facilitate its secretion. Logically, Cba2 is envisaged to be a key player in salvaging and hydrolyzing cellobiose.

The above interpretation helps explain the exceptional performance of Cba2 in converting cellobiose, a strong inhibitor of both CenA and Cex, to a substantially weaker inhibitor, glucose, in filter paper hydrolysis (Holtzapple et al., 1990; Sawant et al., 2021). Moreover, it was previously noted that the hydrolysis of cellotriose by CenA was many times slower than that of cellotetraose (Damude et al., 1996). Other groups also reported that both Eng and Exg were subjected to substantial inhibition by cellodextrins, in particular by the shortest member, cellotriose (Harhangi et al., 2002; Brunecky et al., 2013). Thus, Cba2 might also serve to hydrolyze these oligosaccharides, which could otherwise be present to effectively inhibit both CenA and Cex. From another perspective, as reflected by the relatively long-lasting activity exhibited by Cba2 (Figure 5), it is likely that this cellobiase is a rather durable and glucose-tolerant enzyme (Zhang et al., 2017) which outperforms its competitors during cellulolysis.

Previously, when a Sigma β-glucosidase product prepared from almond was employed to interact with CenA/Cex, only a weak synergistic effect on filter paper hydrolysis was observed (Wong et al., 1988). Among the various factors leading to the unattractive outcome, which could be attributable to either the enzymes or the assay conditions, the lack of a competent cellobiase component(s) to work with CenA/Cex was considered to be the primary reason. In attempting to identify this candidate, we were cautious that the best possible conditions, e.g. higher enzyme dosages (100 U and 2.5 U of CenA and Cex, respectively; Figures 2–6), which might help minimize substrate inhibition (Väljamäe et al., 2001), should be employed. Leveraging a cut-and-try approach and the limited availability of cellobiase samples (0.001–0.01 U) (Figures 2–6), remarkably, we succeeded in picking Cba2 out. Incredibly, Cba2 was shown to be notably outweighed (3–4 times as potent as) its competitors (Figure 5).

Another noteworthy point of Cba2 is its ability to interact with Cba4 to achieve enhanced synergistic effects (Figure 6). Despite working under unoptimized conditions, the filter paper activities obtained from the cooperation between Cba2 and Cba4 (Figure 6) were found to be comparable to those achieved by some commercial cellulase preparations, ranging from 7.7 to 69.9 U ml^−1^ (Yu et al., 2016). To date, although a large number of examples of cooperative action happening between Eng and Exg has been documented (Ghose and Bisaria, 1979; Medve et al., 1998; Duedu and French, 2016), despite the presence of a wide collection of β-glucosidases characterized from a broad spectrum of microbial species, the ability of these enzymes to interact cooperatively among themselves has been rarely reported (Mansfield et al., 1999). Leveraging the application of recombinant bacterial cellulases and the superb performance of Cba2 working either alone or in conjunction with Cba4, our work presented here provides evidence, for the first time, to demonstrate the occurrence of synergies among not only members of different types of cellulases, but also among the less commonly known cellobiases. With Cba2 and Cba4 as working prototypes, research activities may then be focused on their high-level expression, optimization of the working ratios among enzyme participants as well as between enzyme and substrate, and refinement of the conditions optimal for cellulolysis. Once the required particulars are available, Cba2 and Cba4 may prove to be crucial members of a cellulase complex capable of performing cost-effective cellulolysis on a large scale.

## 5 Conclusion

Five cellobiases of a cellulolytic bacterium, *C. biazotea*, were first expressed as recombinant enzymes, with four of them: Cba, Cba3, Cba4, and Cba5 in *E. coli* and the remaining one, Cba2, in *B. subtilis*. When these recombinant cellobiases were employed to interact with another recombinant preparation from *E. coli* comprising CenA and Cex enzymes of *C. fimi*, the five cellobiases were shown to be able to exhibit a positive, but distinctly different intensities of, synergistic effect on filter paper hydrolysis. Notably, Cba2 was found to outperform its rivals by a wide margin of 2–3 times higher in efficiency. More intriguingly, Cba2 was also demonstrated to be able to achieve synergies with another isozyme, Cba4. To our knowledge, our results represent the first demonstration of synergism formed among bacterial cellulases in filter paper hydrolysis. The cooperation between Cba2 and Cba4 may prove to be useful for the formulation of an efficient cellulase complex for cost-effective cellulolysis on a large scale.

## Data Availability

The datasets presented in this study can be found in online repositories. The names of the repository/repositories and accession number(s) can be found in the article/supplementary material.

## Abbreviations

Eng, endoglucanase; Exg, exoglucanase; SN, supernatant; CL, cell lysate.
